# Diverse contributions of MYC2 and EIN3 in the regulation of Arabidopsis jasmonate‐responsive gene expression

**DOI:** 10.1002/pld3.15

**Published:** 2017-10-16

**Authors:** Yuyu Zheng, Yiheng Lan, Tieliu Shi, Ziqiang Zhu

**Affiliations:** ^1^ Jiangsu Key Laboratory for Biodiversity and Biotechnology College of Life Sciences Nanjing Normal University Nanjing China; ^2^ Center for Bioinformatics and Computational Biology Shanghai Key Laboratory of Regulatory Biology Institute of Biomedical Sciences and School of Life Sciences East China Normal University Shanghai China

**Keywords:** Arabidopsis, EIN3, jasmonate, MYC2, transcriptome

## Abstract

Derepression of transcription factors is the key mechanism for triggering plant jasmonate (JA) responses. Unlike regulating certain physiological functions for the majority of transcription factors in JA signaling, MYC2 and EIN3 control more diverse aspects. MYC2 predominantly participates in wounding response, metabolism, and root growth inhibition, while EIN3 (and its closest homolog EIL1) regulates defense gene expression and root hair development. Recently, it was reported that MYC2 and EIN3/EIL1 proteins mutually interact with each other and suppress their interaction partner's transcriptional activities. To understand their contributions in the modulation of transcriptomic network, we initially identified 1,495 differentially expressed jasmonate (JA)‐responsive genes in wild‐type Arabidopsis through RNA‐seq analysis. Among them, 25% or 4.2% were independently regulated by EIN3/EIL1 or MYC2, respectively. Further analysis showed that EIN3/EIL1 and MYC2 interdependently regulate 16.3% of the JA‐regulated transcriptome, including downregulation of three auxin‐related genes, which might confer JA‐inhibited root elongation. Lastly, we found that <30 genes were antagonistically regulated by MYC2 and EIN3/EIL1. We conclude that EIN3/EIL1 play a dominant role while MYC2 largely relies on EIN3/EIL1 for executing its transcriptional activity, either synergistically or antagonistically.

## 1 INTRODUCTION

Jasmonate (JA) is a major plant hormone, which helps plants defend against insect attacks and necrotrophic fungi infections. In addition, JA also regulates a variety of developmental events, including root elongation, root hair density, trichome development, flowering time, male fertility, and senescence (Chini, Gimenez‐Ibanez, Goossens, & Solano, 2016).

In Arabidopsis, there are 13 JASMONATE‐ZIM DOMAIN PROTEINs (JAZs). Without JA, JAZ proteins interact with their downstream transcription factors and suppress their transcriptional activities. Biologically active JA molecules (JA‐isoleucine, JA‐Ile) stimulate the interaction between the F‐box protein CORONATINE INSENSITIVE1 (COI1) and JAZ proteins and lead to JAZ degradation through the 26S proteasome‐mediated protein degradation pathway (Chini et al., 2007; Thines et al., 2007; Yan et al., 2007), thus removing the repressions on transcription factors to elicit various JA responses. Until now, more than a dozen JAZ‐interacting transcription factors have been reported. Most of these transcription factors function in a specific physiological trait. For example, TOE1/TOE2 delays flowering time (Zhai et al., 2015), MYB21/MYB24 are necessary for stamen development (Song et al., 2011), and WRKY57 negatively regulates plant senescence (Jiang, Liang, Yang, & Yu, 2014). In contrast, two branches of JAZ‐interacting transcription factors regulate more diverse aspects. Basic helix‐loop‐helix (bHLH) family transcription factor MYC2, which is the first identified JAZ‐interacting transcription factor, controls wounding response, root growth inhibition, and metabolism (Kazan & Manners, 2013). Plant EIL family transcription factor EIN3 and its closest homolog EIL1, two master regulators in ethylene signaling, interact with JAZ proteins to mediate plant defense responses and root hair growth (Zhu et al., 2011). More interestingly, it has been shown that the transcription of pathogen‐responsive gene *PDF1.2* is induced by EIN3/EIL1 but repressed by MYC2 (Zhu et al., 2011), suggesting the mutual regulation between these two branches of transcription factors. Recently, it was reported that these two groups of transcription factors are able to physically interact with each other and inhibit their interaction partner's transcriptional activity (Song et al., 2014; Zhang et al., 2014), to antagonistically regulate wounding or pathogen‐responsive gene expression (Zhu & Lee, 2015).

**Figure 1:**
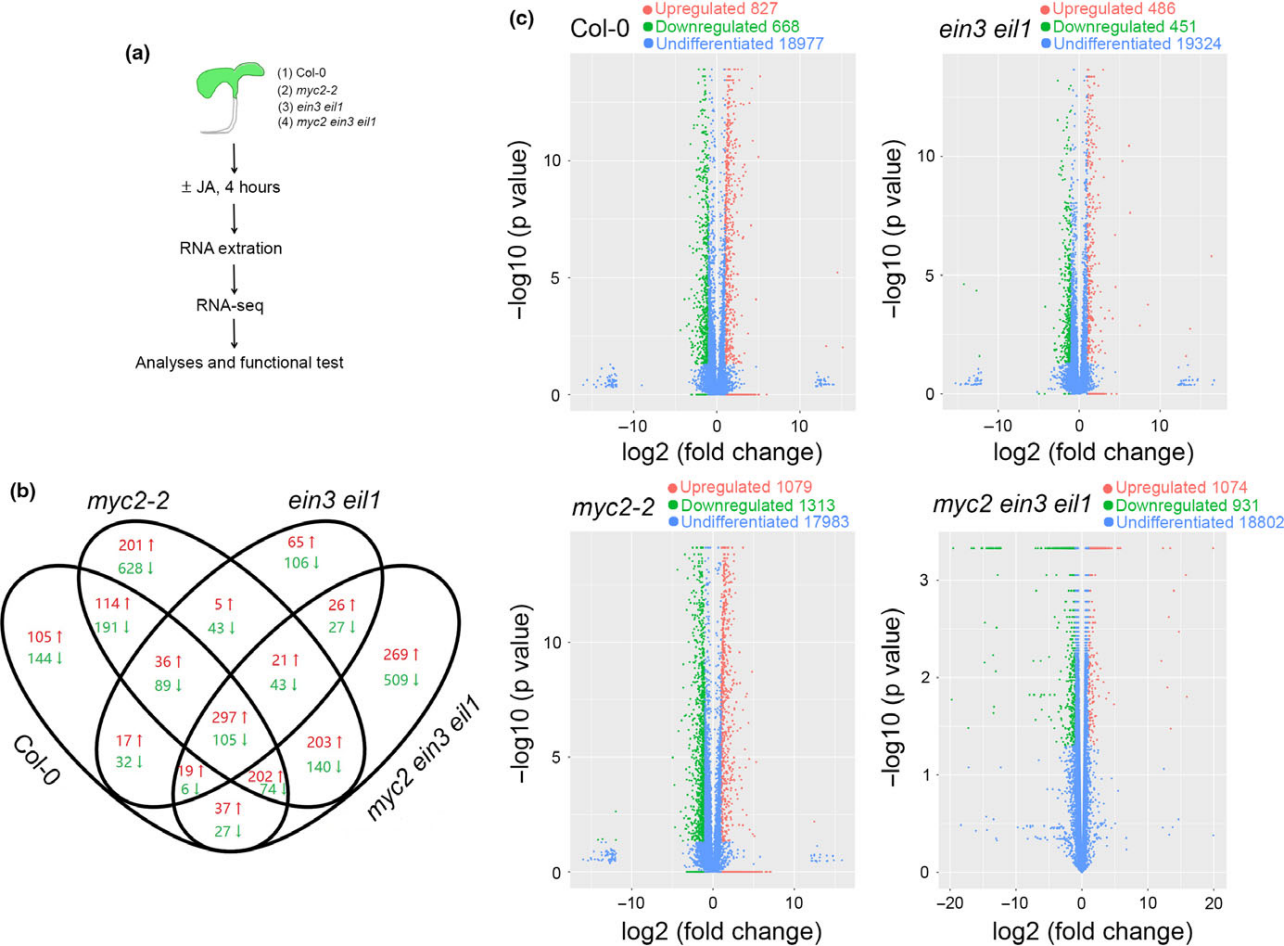
Transcriptomic profiling of JA‐responsive genes. (a) Schematic illustration of experiment design. (b) Venn diagram showing the overlap in differentially expressed genes in each genotype. (c) Differentially expressed genes in each genotype after JA treatment. The exact upregulated, downregulated, or undifferentiated gene numbers were denoted on the top of each panel

Although the MYC2‐regulated transcriptomic profiles have been explored through microarray approach since a decade ago (Dombrecht et al., 2007), it is not clear whether these differentially expressed genes are independently modulated by MYC2, or coregulated with other transcription factors (such as EIN3/EIL1), or antagonistically controlled by MYC2 and EIN3/EIL1. On the other hand, the proportion of the JA‐regulated transcriptome controlled by EIN3/EIL1 has not been determined. To answer these questions and further dissect the relationship between MYC2 and EIN3/EIL1 in controlling the JA‐responsive transcriptome, we checked global gene expression in Col‐0 (wild type), *myc2‐2*,* ein3 eil1,* and *myc2 ein3 eil1* mutants with or without JA treatment (Figure 1a).

## 2 MATERIALS AND METHODS

### 2.1 Plant materials and growth conditions

All the mutants used in this study are in Col‐0 background. The seeds of *myc2‐2* (Zhu et al., 2011), *ein3 eil1* (Zhu et al., 2011), *myc2 ein3 eil1* (Zhang et al., 2014), and *coi1‐2* (Zhu et al., 2011) have been described previously. Seeds were sterilized in 10% bleach and 0.1% Triton X‐100 for 5 min, washed five times with sterile water, and then placed on solid MS medium (4.4 g/L Murashige and Skoog Basal salt, 10 g/L sucrose, 8 g/L agar, pH = 5.8). After 3 days of stratification, plates were placed at 22°C for growth (80/μmolm^2^/s^1^ continuous white light). Methyl jasmonate (Sigma‐Aldrich) stock solution (100 mM, dissolved in ethanol) was prepared and used as the bioactive JA for treatment in this study. For RNA‐seq or gene expression analysis, about 200 6‐day‐old light‐grown seedlings were soaked in 100 μM JA (widely used treatment concentration) (Dombrecht et al., 2007; Liu et al., 2016; Song et al., 2014) or Mock (same concentration of solvents) for 4 hrs. Then, seedlings were harvested and immediately stored in liquid nitrogen for subsequent RNA extraction. For root length recording, seedlings were grown vertically on various concentrations of JA medium for 1 week and then analyzed. At least thirty individual seedlings were measured for each treatment. One‐way ANOVA post hoc Tukey test was used for calculating statistical significance.

### 2.2 RNA‐seq and data processing

After treatment, samples were collected and immediately stored in liquid nitrogen for RNA extraction. Each sample has three independent biological replicates. Total RNAs were extracted using the Spectrum Plant Total RNA Kit (Sigma‐Aldrich). Library construction and sequencing were performed in Vazyme Biotech Co., Ltd (Nanjing, China). Briefly, RNA quantity was analyzed by Bioanalyzer 2100 (Agilent), and RNAs with RIN > 8.0 were used for RNA‐seq. Approximately 3 μg of total RNA was used to isolate poly‐(A) mRNA with poly‐T oligo‐attached magnetic beads (Invitrogen). Following purification, the mRNA was fragmented into small pieces by adding fragmentation buffer. Then, the cleaved RNA fragments were reverse‐transcribed to create the final cDNA library in accordance with the protocol for the mRNA‐Seq sample preparation kit (Illumina). The average insert size for the paired‐end libraries was 300 bp. Paired‐end sequencing (100 bp) was carried out on HiSeq2500 platform (Illumina). The sequenced raw data were filtered to remove low‐quality tags (reads with unknown nucleotides “N”), empty reads (no read sequence between the adaptors), and reads with only one copy number (reads that might have resulted from sequencing errors). RNA‐seq reads were mapped to the reported *Arabidopsis thaliana* genome (version TAIR 10) using TopHat with standard parameters (Trapnell, Pachter, & Salzberg, 2009). The number of reads mapped to each annotated Arabidopsis gene in TAIR10 was determined using HTSeq‐count (Anders, Pyl, & Huber, 2015), and then, the RPKM expression ratios (fold changes) of JA‐treated and control samples were subject to a regular log‐transformation, implemented in the DESEQ2 package (Love, Huber, & Anders, 2014). In our analysis, genes were considered to be significantly differentially regulated if they had | log2 (fold change) | > 1 and *p* value < .05 with the Benjamin and Hochberg correction for multiple testing in a given pair of treatments.

### 2.3 Transcriptome analysis

The classifications for EIN3/EIL1‐ and MYC2‐regulated genes were based on multiple comparison strategies. The genes independently regulated by EIN3/EIL1 were determined with the criteria: upregulated or downregulated in both Col‐0 and *myc2* but not altered in *ein3 eil1* or *myc2 ein3 eil1* (| log_2_ $\left(\frac{\mathrm{WT},\mathrm{JA}}{\mathrm{WT},\mathrm{Mock}}\right)$ | > 1, | log_2_ $\left(\frac{\mathrm{myc}2,\;\mathrm{JA}}{\mathrm{myc}2,\;\mathrm{Mock}}\right)$ | > 1, | log_2_ $\left(\frac{\mathrm{myc}2,\;\mathrm{JA}}{\mathrm{WT},\mathrm{JA}}\right)$ | < 1, | log_2_ $\left(\frac{\mathrm{ein}3\;\mathrm{eil}1,\;\mathrm{JA}}{\mathrm{ein}3\;\mathrm{eil}1,\;\mathrm{Mock}}\right)$ | < 1, and | log_2_ $\left(\frac{\mathrm{myc}2\;\mathrm{ein}3\;\mathrm{eil}1,\;\mathrm{JA}}{\mathrm{myc}2\;\;ein3\;\mathrm{eil}1,\;\mathrm{Mock}}\right)$ | < 1). Similarly, the genes independently regulated by transcription factor MYC2 were identified with the condition of | log_2_ $\left(\frac{\mathrm{WT},\mathrm{JA}}{\mathrm{WT},\mathrm{Mock}}\right)$ | > 1, | log_2_ $\left(\frac{\mathrm{ein}3\;\mathrm{eil}1,\;\mathrm{JA}}{\mathrm{ein}3\;\mathrm{eil}1,\;\mathrm{Mock}}\right)$ | > 1, | log_2_ $\left(\frac{\mathrm{ein}3\;\mathrm{eil}1,\;\mathrm{JA}}{\mathrm{WT},\mathrm{JA}}\right)$ | < 1, | log_2_ $\left(\frac{\mathrm{myc}2,\;\mathrm{JA}}{\mathrm{myc}2,\;\mathrm{Mock}}\right)$ | < 1, and | log_2_ $\left(\frac{\mathrm{myc}2\;\mathrm{ein}3\;\mathrm{eil}1,\;\mathrm{JA}}{\mathrm{myc}2\;\mathrm{ein}3\;\mathrm{eil}1,\;\mathrm{Mock}}\right)$ | < 1. The genes synergistically regulated by both MYC2 and EIN3/EIL1 were determined with this criteria: Expression level only changed in Col‐0 but not in either *myc2*,* ein3 eil1,* or *myc2 ein3 eil1* (| log_2_ $\left(\frac{\mathrm{WT},\mathrm{JA}}{\mathrm{WT},\mathrm{Mock}}\right)$ *|* > 1*, |* log_2_ $\left(\frac{\mathrm{myc}2,\;\mathrm{JA}}{\mathrm{myc}2,\;\mathrm{Mock}}\right)$ *|* *<* 1*, |* log_2_ $\left(\frac{\mathrm{ein}3\;\mathrm{eil}1,\;\mathrm{JA}}{\mathrm{ein}3\;\mathrm{eil}1,\;\mathrm{Mock}}\right)$ *|* < 1, and | log_2_ $\left(\frac{\mathrm{myc}2\;\mathrm{ein}3\;\mathrm{eil}1,\;\mathrm{JA}}{\mathrm{myc}2\;\mathrm{ein}3\;\mathrm{eil}1,\;\mathrm{Mock}}\right)$ *|* < 1). The EIN3/EIL1‐induced but MYC2‐inhibited genes were obtained based on following criteria: Expression levels were upregulated in Col‐0 but not altered in either *ein3 eil1* or *myc2 ein3 eil1;* meanwhile, these genes were induced in *myc2* mutant with higher amplitude (log_2_ $\left(\frac{\mathrm{WT},\mathrm{JA}}{\mathrm{WT},\mathrm{Mock}}\right)$ > 1*,* log_2_ $\left(\frac{\mathrm{myc}2,\;\mathrm{JA}}{\mathrm{WT},\mathrm{JA}}\right)$ > 1*,* log_2_ $\left(\frac{\mathrm{ein}3\;\mathrm{eil}1,\;\mathrm{JA}}{\mathrm{WT},\mathrm{JA}}\right)$  < *−*1*, |* log_2_ $\left(\frac{\mathrm{ein}3\;\mathrm{eil}1,\;\mathrm{JA}}{\mathrm{ein}3\;\mathrm{eil}1,\;\mathrm{Mock}}\right)$ *|* < 1*, and |* log_2_ $\left(\frac{\mathrm{myc}2\;\mathrm{ein}3\;\mathrm{eil}1,\;\mathrm{JA}}{\mathrm{myc}2\;\mathrm{ein}3\;\mathrm{eil}1,\;\mathrm{Mock}}\right)$ *|* < 1). Similarly, the MYC2‐induced but EIN3/EIL1‐inhibited genes were determined by the criteria of log_2_ $\left(\frac{\mathrm{WT},\mathrm{JA}}{\mathrm{WT},\mathrm{Mock}}\right)$ > 1*,* log_2_ $\left(\frac{\mathrm{ein}3\;\mathrm{eil}1,\;\mathrm{JA}}{\mathrm{WT},\mathrm{JA}}\right)$ > 1*,* log_2_ $\left(\frac{\mathrm{myc}2,\;\mathrm{JA}}{\mathrm{WT},\mathrm{JA}}\right)$  < *−*1*, |* log_2_ $\left(\frac{\mathrm{myc}2,\;\mathrm{JA}}{\mathrm{myc}2,\;\mathrm{Mock}}\right)$ *|* < 1, and | log_2_ $\left(\frac{\mathrm{myc}2\;\mathrm{ein}3\;\mathrm{eil}1,\;\mathrm{JA}}{\mathrm{myc}2\;\mathrm{ein}3\;\mathrm{eil}1,\;\mathrm{Mock}}\right)$ *|* < 1.

### 2.4 Gene Ontology (GO) enrichment analyses

We performed GO enrichment analysis for different clusters of EIN3/EIL1‐ and MYC2‐regulated genes with the bioinformatic resource DAVID 6.8 (Huang da, Sherman, & Lempicki, 2009a,b), which retrieves the GO terms for the gene models using the structured vocabulary provided by the Gene Ontology project (http://www.geneontology.org), and selected the enriched GO terms using the parameter of fold enrichment >1 and *p* value <.05 (a modified Fisher's exact *p* value). GO enrichment analysis associates each gene of a list of different biological processes and then evaluates whether the list contains more genes than expected “by chance” for a certain biological process.

### 2.5 RNA expression and quantitative real‐time PCR (qRT‐PCR)

Total RNA was extracted using TRIzol reagent (Invitrogen). After genomic DNA removal, at least one microgram of RNA was reverse‐transcribed into cDNA using the PrimeScript RT reagent kit (TaKaRa). Real‐time PCRs were carried out on a LightCycler 96 PCR machine (Roche) with SYBR Premix Ex Taq enzyme (TaKaRa). All the primers used for qRT‐PCRs were listed inTable  S6.

## 3 RESULTS

More than 90% of each sample's sequencing reads were mapped to the *Arabidopsis* genome (TAIR 10), and more than 88% of reads were uniquely mapped to a single location (Table S1), suggesting that our RNA‐seq results are reliable. We identified 1,495 genes (827 upregulated genes; 668 downregulated genes) that were differentially expressed after JA treatment in Col‐0 with an absolute fold change ≥2 and *p* value < .05 selection criteria (Figure 1b–c). With the same criteria, 2,392 genes (1,079 upregulated genes and 1,313 downregulated genes) and 937 genes (486 upregulated genes and 451 downregulated genes) were found to be differentially expressed in *myc2* and *ein3 eil1* mutants, respectively (Figure 1b–c). In *myc2 ein3 eil1* triple mutants, 2,005 (1,074 upregulated genes and 931 downregulated genes) genes were differentially expressed (Figure 1b–c). We then randomly selected ten genes from each datasheet and performed quantitative reverse transcription‐PCR (qRT‐PCR) to verify the expression changes. Most of the qRT‐PCR results were comparable to our RNA‐seq data (Fig. S1). The complete list of differentially expressed genes is presented in Table S2.

**Figure 2:**
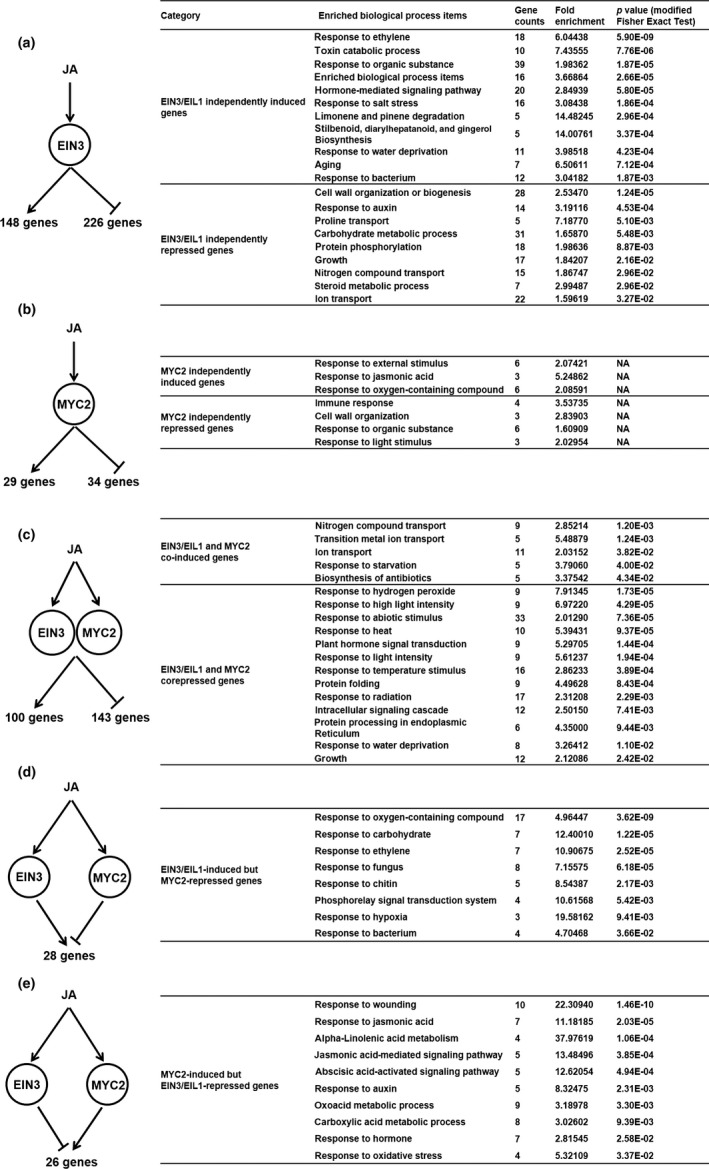
Overview of five regulating models of MYC2 and EIN3/EIL1 in JA signal pathway. (a) EIN3/EIL1 independently regulating model and GO enrichment analysis. (b) MYC2 independently regulating model and GO enrichment analysis. (c) MYC2 and EIN3/EIL1 synergistically regulating model and GO enrichment analysis. (d) EIN3/EIL1‐induced but MYC2‐repressed regulating model and GO enrichment analysis. (e) MYC2‐induced but EIN3/EIL1‐repressed regulating model and GO enrichment analysis. For each panel, arrowhead represents gene inducing process; blunt head indicates repression. The enriched biological process terms shown for each regulating model represent the mainly significant functions for the regulated genes. Fold enrichment means the ratio of actual gene number and expected gene number in a given GO term; *p* values were evaluated via a modified Fisher's exact test. In particular, the *p* values in MYC2 independently regulating model were missing (NA) due to the limitation of statistical significance and low‐number genes

We then did a series of comparisons to answer the next five questions based on our RNA‐seq results. Firstly, what kinds of genes are upregulated or downregulated only by EIN3/EIL1 upon JA treatment? These genes should fit the following two criteria: (i) upregulated or downregulated in both Col‐0 and *myc2*; (ii) not altered in *ein3 eil1* or *myc2 ein3 eil1*. We found that 148 genes were EIN3/EIL1 independently induced and 226 genes were EIN3/EIL1 independently repressed (Figure 2a and Table S3). Gene ontology (GO) analysis showed that most of these induced genes were enriched in ethylene response, abscisic acid response, salt stress, or water deprivation response, whereas repressed genes were mainly involved in cell wall organization, auxin response, nitrogen, and ion transport.

Secondly, we sought to identify MYC2 independently regulated genes. With a comparable strategy, we selected genes that were induced or repressed only in Col‐0 and *ein3 eil1* but not changed in *myc2* or *myc2 ein3 eil1* mutants. We only got 63 genes as MYC2 independently regulated genes (29 genes were upregulated and 34 genes were downregulated) (Figure 2b and Table S3). GO analysis showed that MYC2 independently upregulated genes were enriched in JA response and downregulated genes focused on immune response and light response. This result is consistent with previous reports showing that MYC2 represses pathogen‐responsive gene expression (Zhu et al., 2011) and blue light‐mediated photomorphogenesis (Yadav, Mallappa, Gangappa, Bhatia, & Chattopadhyay, 2005).

**Figure 3:**
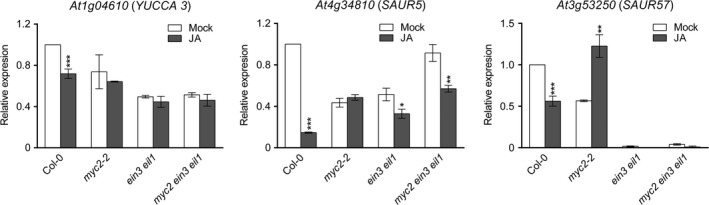
Auxin‐related gene expression. qRT‐PCR to determine gene expression levels in 6‐day‐old seedlings treated with 100 μM JA or Mock for 4 hrs

**Figure 4:**
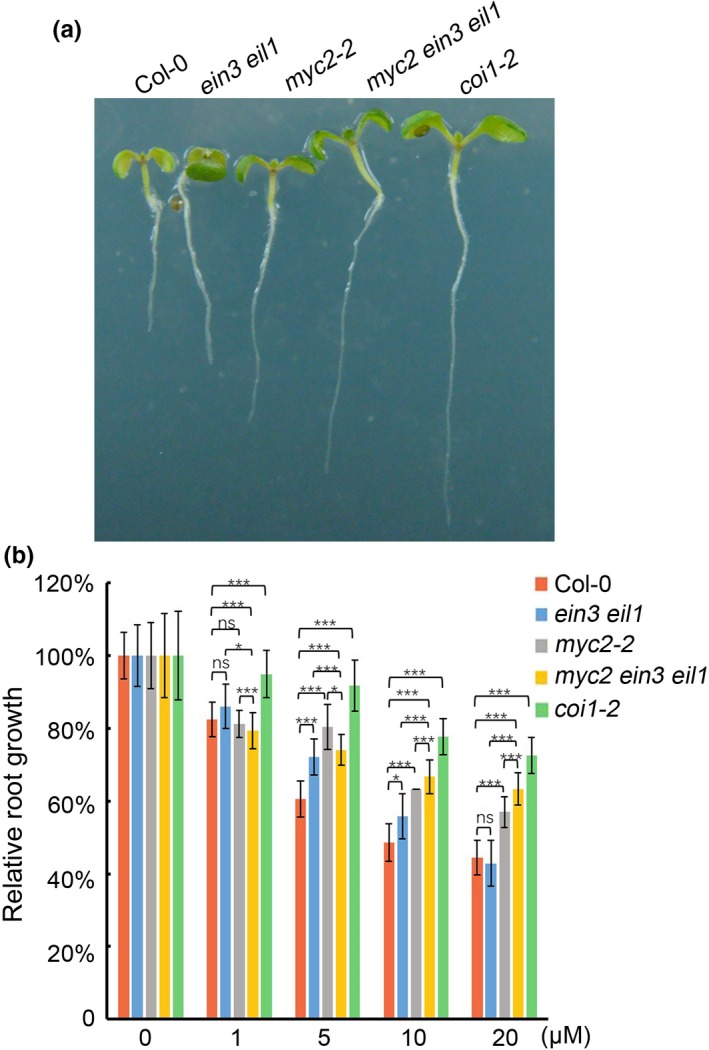
Root length phenotype. (a) Representative 7‐day‐old seedling grown on 10 μM JA was imaged. (b) Relative root growth inhibition of 7‐day‐old seedlings grown on various concentrations of JA was analyzed. Root length grown in the presence of various concentration of JA was normalized with no JA control. The weak allele of JA receptor mutant (*coi1‐2*) was included as a control to show the JA insensitivity

Thirdly, we examined genes that are synergistically regulated by both MYC2 and EIN3/EIL1. We selected genes differentially expressed in only Col‐0 but not in either *myc2*,* ein3 eil1,* or *myc2 ein3 eil1* background and totally found 243 genes (100 genes were upregulated, and 143 genes were downregulated) (Figure 2c and Table S4). GO analysis indicated that genes related to nitrogen compound transport, metal ion transport, and starvation response were enriched in MYC2 and EIN3/EIL1 synergistically upregulated categories. However, gene response to external stimuli (high light or heat) or endogenous hormones appeared in the downregulated category. We noticed that the auxin biosynthesis gene (*YUCCA3*) and light/auxin‐responsive genes (*SAUR5* and *SAUR57*) were negatively regulated by MYC2 and EIN3/EIL1 in our RNA‐seq result and further confirmed this result by qRT‐PCR (Figure 3). It has been shown that MYC2 reduces *PLETHORA1* (*PLT1*) and *PLT2* expressions to negatively regulate auxin‐mediated root stem cell niche maintenance and root elongation (Chen et al., 2011). Given that MYC2 and EIN3/EIL1 synergistically reduce auxin‐related gene expression, we then tested root elongation response and found that *myc2 ein3 eil1* was less sensitive to JA, comparing with *myc2* or *ein3 eil1* parental lines (Figure 4). We conclude that MYC2 and EIN3/EIL1 inhibit root elongation likely through the reduction in other auxin‐related gene expression.

Because biochemical studies already show that MYC2 and EIN3/EIL1 mutually inhibit each other's activity, we then explored the number of genes that are antagonistically regulated by these transcription factors. To find EIN3/EIL1‐induced but MYC2‐inhibited genes, we selected genes whose expression levels were upregulated in Col‐0 but not altered in either *ein3 eil1* or *myc2 ein3 eil1;* meanwhile, these genes should also be induced in *myc2* mutant with a higher amplitude. We uncovered 29 genes that fit these criteria (Figure 2d and Table S5) and found that these genes are mainly involved in response to pathogens (fungus or bacterium), ethylene, or hypoxia stress.

Lastly, with similar criteria, we identified 26 genes which were induced by MYC2 but suppressed by EIN3/EIL1 (Figure 2e and Table S5). These genes are enriched in wounding response, JA response, and metabolic processes. Our transcriptional analysis successfully recaptured the published MYC2 and EIN3/EIL1 antagonistically regulated gene categories.

With above‐mentioned comparisons, we conclude that EIN3/EIL1 and MYC2 act in concert to regulate a proportion of JA responses. EIN3/EIL1 themselves regulate one‐quarter of JA‐responsive transcriptome that involve hormone cross talk, abiotic stress responses, and cell wall organization, whereas MYC2 cooperates with EIN3/EIL1 to modulate many wounding and metabolic‐related genes. Our study also finds that MYC2 and EIN3/EIL1 negatively regulate auxin synthesis or responsive genes and contribute to root growth inhibition.

## 4 DISCUSSION

We reveal five distinct regulatory categories for MYC2 and EIN3/EIL1 in modulating JA responses but raise some new questions: (i) How many genes are directly regulated by MYC2 or EIN3/EIL1? (ii) What is the molecular basis for MYC2 and EIN3/EIL1 transcriptome regulation either synergistically or antagonistically? We believe that future chromatin immunoprecipitation (ChIP)‐seq will help to answer these questions. In addition, because both MYC2 and EIN3/EIL1 interact with Mediator component (Med25) (Chen et al., 2012; Yang et al., 2014), it is plausible to test whether these transcription factors will compete with Mediator complex for regulating transcription. Moreover, the spatial and temporal expression pattern of MYC2 and EIN3/EIL1 themselves should be considered for further understanding their physiological roles.

It has been reported that MYC3/MYC4/MYC5 act redundantly with MYC2 in the control of JA responses (Cheng et al., 2011; Fernandez‐Calvo et al., 2011; Niu, Figueroa, & Browse, 2011; Qi, Huang, Song, & Xie, 2015; Qi, Wang, et al., 2015). Although we concluded that MYC2 played lesser roles than EIN3/EIL1 in the JA‐regulated transcriptome, it is plausible that these transcription factors compensated for the lack of MYC2 activity in the *myc2* mutant used in our experiment and thus limited the resolution of the experiment. It may be necessary to characterize these MYC2 homolog proteins (MYC3/MYC4/MYC5) in the future to fully understand *MYC2* and its relative gene functions.

Finally, it will be of interest to analyze relationships among other JAZ‐interacting transcription factors. For example, it was recently reported that MYC2 can also interact with MYB21/MYB24 to cooperatively regulate stamen development (Qi, Huang, *et al*., 2015; Qi, Wang, et al., 2015) and antagonize another group of bHLH transcription factors in leaf senescence (Qi, Huang, *et al*., 2015; Qi, Wang, et al., 2015). Our RNA‐seq result also showed that JA could still differently regulate 2,005 gene expression in *myc2 ein3 eil1* (Figure 1c), even more than in wild‐type background. This result suggests that plenty of genes would be antagonistically regulated by MYC2/EIN3/EIL1 and other transcription factors. It is not surprising that these cross‐family JAZ‐interacting transcription factors interact with each other and coordinate plant growth and development for adapting to environmental changes (Zhu & Lee, 2015).

## ACCESSION NUMBER

The transcriptome datasets are available at the NCBI Sequence Read Archive (https://www.ncbi.nlm.nih.gov/sra) with accession number SRP092587.

## CONFLICT OF INTEREST

The authors declare no conflict of interest.

## AUTHOR CONTRIBUTION

T.S. and Z.Z. designed the research. Y.Z. and Z.Z. carried out experiments. Y.L. and T.S. performed bioinformatics analysis. Z.Z. wrote the manuscript. All authors analyzed data.
