# Supplementary material for: Diverse contributions of MYC2 and EIN3 in the regulation of Arabidopsis jasmonate‐responsive gene expression
Source: Plant Direct. 2017 Oct 16;1(4):e00015. doi: 10.1002/pld3.15 (PMC6508547; doi:10.1002/pld3.15)
Supplement: Supplementary file 1 [file PLD3-1-e00015-s001.pdf]

Supplementary Figure

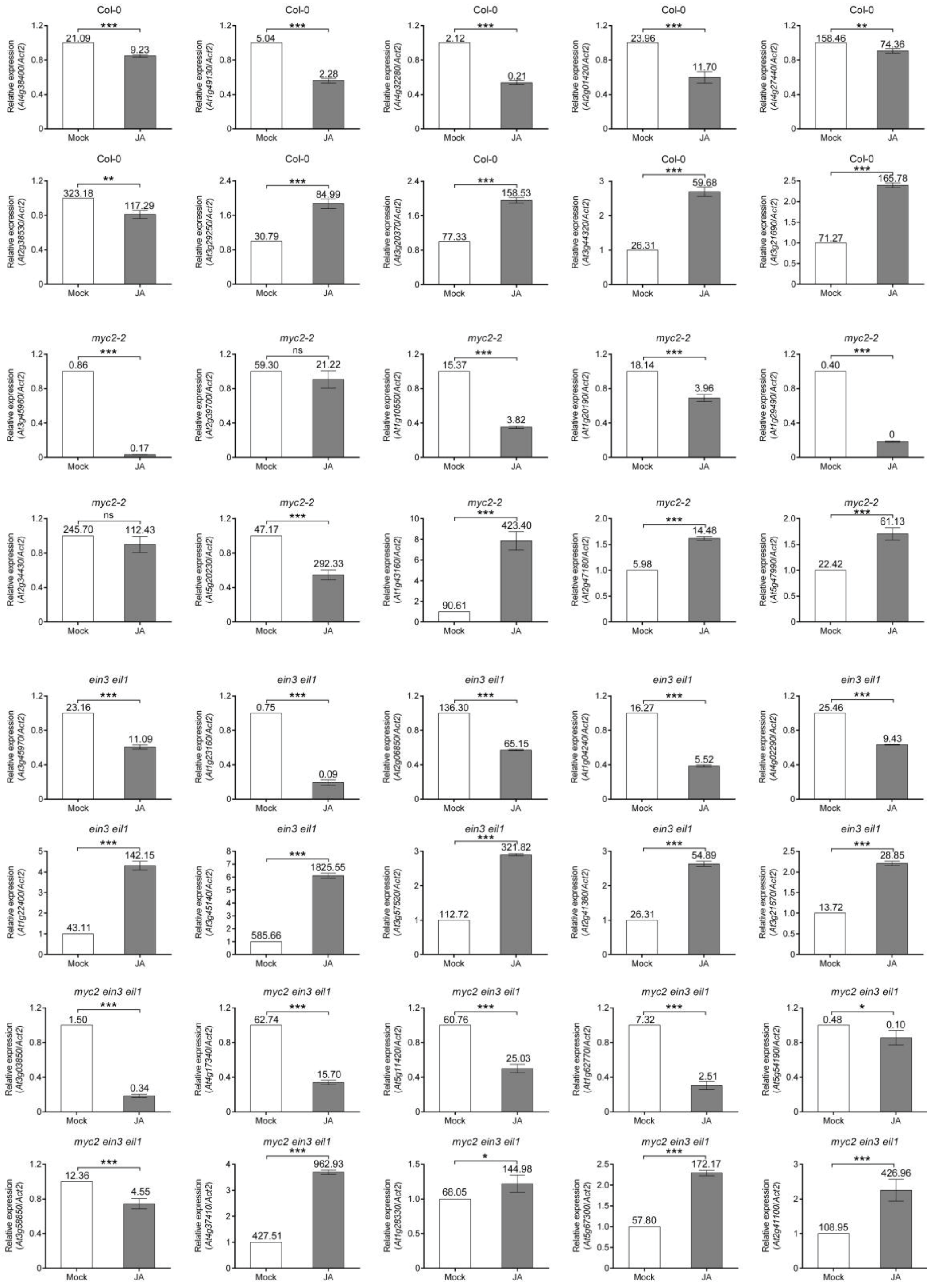

Figure 1. qRT-PCR assays to verify RNA-seq results

The expression levels of randomly selected genes from each genotype were checked by qRT-PCR. The statistical significance for qRT-PCR results were calculated and marked. To compare qRT-PCR with RNA-seq results, the FPKM values from the original RNA-seq data were directly presented above of each bar.
